# The Relation of Dentistry and Medicine

**Published:** 1887-04

**Authors:** Walter A. Dun


### The Relation of Dentistry and Medicine.

BY WALTER A. DUN, M.D., M. R. C. S.

[Read at the Forty-third Annual Meeting of the Mississippi Valley Dental Association.]

To speak of the relation of these branches to each other, is to speak of the relation of a part to the whole, or of a child to its mother. When medicine is taken in its broadest sense as embracing everything which pertains to the practice of the healing art, the science of dentistry is clearly embraced within its scope.

As knowledge advanced in the broad art of healing, three great branches, surgery, obstetrics, and general medicine crawled about in the dark ignorance which surrounded the old parent, as the most clearly defined subjects, with broad differences to distinguish them as the early offspring. In the historical aspects of these births there is much that seems ludicrous. Thus the subject of surgery not only included dentistry, but the barber's art, and in general drifted in that way. While general medicine got tinctured with monks and their ways, and thus in the early start upon their separate roads, one was accompanied with the rough usage, and coarse, heartless ways of the barbers, whose very name conveys what barbarians they were and are, while general medicine took the way with peaceful followers and teachers of Christ, and thus become impressed with a divine nature to her calling. There were many offshoots in the past from all these great divisions, but from none have so many sprung as from surgery, or as it was styled before the barbers and surgeons split, "barbary." Naturally from old association, some of the odors, and perchance some salves of the barbers clung to the surgeons, so that an offensive odor fell to surgery in the start, which it has striven long and manfully to throw off. A difficult undertaking when we all recall how seemingly brutal and unfeelingly bloody operations appear to-day. Let us only imagine then how much more brutal many of them were, and how painful they appeared in the long experimental evolution of that art before the days of anæsthetics. Do we wonder that from such a branch any subdivision was loth to separate, and endeavor to lead a respectable course and leave nauseous odors and associations of the older division behind?

Dentistry is one of these divisions of medicine then, more closely speaking, a subdivision of that branch of medicine called surgery, which left the surgeons ranks a long time ago and has probably strayed further from the original source than any other division. Dentistry, however, in common with many other branches of surgery, has grown into a distinct and well recognized specialty. It has relations to both surgery and general medicine, but a long separation has seemed to estrange it almost completely from both. In recent years the tendency is to a closer relation. The members of both professions have come to recognize the importance of a closer understanding. That there are mutual benefits to be derived from a better feeling is certain, for the use of chloroform and antiseptics in the past, shows what interchange of useful remedies of the one will do for the other. These relations which I have just been speaking of apply more closely to surgery, with which the relations of dentistry are obviously many. It is my present purpose, in a short paper, to point out some relations between general medicine and dentistry which I believe are not sufficiently recognized and dwelt upon in both professions, and probably these are not as widely known in the profession of medicine as they should be.

Before entering upon the subject matter of this paper, I must congratulate the dental profession upon their success and the high recognition which they have gained for themselves. I have already pointed out the barbarous odors which surround their birth. These have not been without additions from foul mouths and breaths. Yet, with all the inherited and accumulated repugnance in the practice of dentistry, you have so conducted yourselves and improved and advanced your calling, by wondrous progress, that all the bad caste of dentistry is lost in the recognition of your enlightenment and attainments. I congratulate you again upon the recognition which you justly deserve, and which you have compelled all alike to grant. It is with no little pride, too, that I claim most of this for America and the Americans. The dentists have outstripped the American physicians in keeping the lead of their profession throughout the world, and have reason to be justly proud of it.

From quite a number of points of relationship between dentistry and medicine I have selected two, which are far more important than all others, viz: troubles with dyspepsia and hypnotism.

The teeth are the most important index to the proper food for a child. When they appear, their presence means that they have come to be used, and in proportion to their number and grouping are we justified in changing the diet from the milk, which is supplied from the breast of the mother, to the other articles of adult diet which require chewing and grinding in order to prepare them for their normal solution by the juices of the alimentary canal. Most of our great mortality among infants arises from improper food, and not paying attention to the teeth, which stand in the mouth as *Nature's* index to guide all.

In adult life, from the various factors which may be summed up as factors of civilization, the teeth often decay early and become imperfect. It too often happens that from the rush of strife in business, more active in the adult period than at any other of life, the teeth are allowed to go unkept, unthought of, and almost unused. The neglect of these brings foulness and decay, which pollutes the air we breath and food we swallow. The work of the teeth is thrown on the stomach—to which the foulness of the former is added—and instead of wholesome, clean, very finely masticated food, which the stomach should normally receive, it gets a mass of large lumps foul with large colonies of micro-organisms and decomposing remnants of mucus and food, dislodged from the teeth. Dyspepsia in all its varieties and stages results. In case the total disregard of natural laws of the teeth continues, pyrosis, acid stomach, or water-brash arises to complete the destruction by chemical solution of the molars which decay has spared. The condition of dyspepsia, associated with imperfect mastication, is so common in my experience that I have yet to see a single case of dyspepsia without it.

The medical treatment of dyspepsia in nearly all early cases is successful in case the patients will resume the normal use of the teeth, while all chronic cases are greatly relieved by this plan alone. In old age, where from use or age the teeth are imperfect or lost, there is a return to the condition of liquid food of infancy, still the depressed vitality of old age, and the taste for the diet acquired in adult life, brings indigestion in its train, which it has been the province of dentistry to relieve by artificial teeth. Artificial teeth are a greater boon to mankind than glasses for the eyes, and the day is near when to wear them will be as much the fashion and as respectable as glasses are to-day.

The medical profession is too apt to forget the teeth and mastication and resort to many other means. Dentists on the other hand have, in a measure, been led into artificicial teeth by the vanity of the looks of the wearer, yet they have succeeded in perfecting a most important aid to all physicians in the treatment of indigestions. By these means the folly of adult life can be partially relieved and the period of liquid food in old age put off many years. The follies of men are always to be with us; they invariably choose the pound of cure to the ounce of prevention, consequently in preserving the teeth, or supplying their places with artificial substitutes, dentists give physicians great means for the cure of dyspepsias, and alleviating the suffering of advanced life, which physicians ought to be the first to appreciate and advocate.

All I have said about dyspepsias has been to show how much the profession of medicine owes to modern dentistry. In the second point *hypnotism*, I desire to point out to this association of dentists that medicine has been a power which can often be turned to their advantage. This art of hypnotism is strongly urged by Charcot, and while as old as the hills, has for a long time been considered a dark art, and unfortunately poohpooed by most men as savoring of quackery and charlatanism. I predict that a few years will find this useful art and psychic power widely diffused and practiced for the relief of pain in filling and extracting teeth. Only remember, that in its use you need practice, skill, and manliness, and you will find it an invaluable power. For local anæsthetic effect it does as well as chloroform, and is accompanied with infinitely less danger.

